# Bayesian network meta-analysis of the effects of different cognitive interventions on cognitive function in older adults with cognitive impairment

**DOI:** 10.3389/fpubh.2026.1820948

**Published:** 2026-08-11

**Authors:** Yumeng Gong, Juanjuan Wang, Xiaoshuang Zhang, Dongyu Hu, Jiajia Chen, Xiumu Yang, Yongli Duan

**Affiliations:** 1 Fuyang People’s Hospital, Fuyang, Anhui, China; 2 School of Nursing, Bengbu Medical University, Bengbu, Anhui, China

**Keywords:** Bayesian network meta-analysis, cognitive function, cognitive intervention, geriatric cognitive impairment, randomized controlled trial

## Abstract

**Objective:**

This Bayesian network meta-analysis evaluates the comparative efficacy of cognitive interventions on cognitive function in elderly individuals with cognitive impairment.

**Methods:**

Randomized controlled trials examining the effects of cognitive interventions on cognitive function in older adults aged 60 years and above with cognitive impairment were searched in the Cochrane Library, PubMed, Embase, Web of Science, China National Knowledge Infrastructure (CNKI), China Biomedical Literature Service System (SinoMed), VIP Database, and Wanfang Data. The search covered the period from database inception to August 2025. Two researchers independently performed study selection, data extraction, and risk-of-bias assessment using the Cochrane risk-of-bias tool. RevMan 5.4 was used for risk-of-bias visualization, Stata 16.0 was used to construct the evidence network plots, and Bayesian network meta-analysis was conducted using the gemtc package in R version 4.5.2.

**Results:**

This meta-analysis incorporated 35 randomized controlled trials (RCTs), encompassing a total of 2,316 patients. Eight distinct cognitive intervention modalities were evaluated. Regarding the Mini-Mental State Examination (MMSE) scores, surface under the cumulative ranking curve (SUCRA) analysis revealed the following probability ranking for efficacy: computerized cognitive training (CCT; SUCRA = 81.08%), non-invasive brain stimulation (NIBS; SUCRA = 73.87%), cognitive stimulation therapy (CST; SUCRA = 67.24%), serious games (SUCRA = 65.64%), memory training (SUCRA = 61.79%), integrated cognitive training (SUCRA = 51.87%), virtual reality (VR; SUCRA = 27.02%), usual care (SUCRA = 14.38%), and olfactory training (OT; SUCRA = 7.10%). League table comparisons further demonstrated statistically significant improvements in MMSE scores compared to control groups for several interventions: CCT (mean difference [MD] = 2.14, 95% CI: 0.90 to 3.39), integrated cognitive training (MD = 1.28, 95% CI: 0.24 to 2.31), CST (MD = 1.70, 95% CI: 0.80 to 2.65), and serious games (MD = 1.67, 95% CI: 0.23 to 3.13). For the Montreal Cognitive Assessment (MoCA) scores, the SUCRA ranking was: memory training (SUCRA = 70.92%), CST (SUCRA = 70.63%), CCT (SUCRA = 69.71%), integrated cognitive training (SUCRA = 60.27%), virtual reality (VR; SUCRA = 48.24%), serious games (SUCRA = 46.16%), NIBS (SUCRA = 29.93%), and usual care (SUCRA = 4.15%). Significant enhancements in MoCA scores versus control were observed for: CCT (MD = 3.13, 95% CI: 1.79 to 4.50), integrated cognitive training (MD = 2.81, 95% CI: 1.41 to 4.28), and CST (MD = 3.26, 95% CI: 1.16 to 5.50).

**Conclusion:**

Current evidence suggests that CCT, CST, memory training, and NIBS may be more effective interventions for improving cognitive function in elderly individuals with cognitive impairment.

## 1 Introduction

As global population ageing continues to accelerate, the prevalence of cognitive impairment and dementia is also increasing, with the number of affected individuals projected to exceed 152 million by 2050 (1). Cognitive impairment refers to multidimensional deficits in cognitive functioning caused by abnormal brain function or neurodegenerative changes and may involve memory, attention, language, executive function, orientation, visuospatial ability, and information-processing speed (2). It is frequently accompanied by behavioural changes, reduced environmental adaptability, and psychological problems such as depression and anxiety (3). These consequences not only substantially impair the quality of life of older adults but also increase their risk of home-related injuries, including falls and burns (4, 5). Therefore, identifying effective cognitive interventions to improve cognitive function in older adults with cognitive impairment has become an important priority in current and future healthcare. Current clinical management strategies can be broadly classified into pharmacological and non-pharmacological interventions. Although pharmacological treatments, particularly cholinesterase inhibitors and memantine, may provide modest improvements in cognitive symptoms, their overall efficacy remains limited. Moreover, adverse effects such as nausea, vomiting, diarrhoea, loss of appetite, dizziness, and headache may compromise long-term treatment adherence (6, 7). In contrast, non-pharmacological interventions have received increasing attention because of their relatively low risk, limited adverse effects, and high acceptability (8). These interventions encompass a wide range of approaches, including computerized cognitive training, integrated cognitive training, virtual reality-based training, cognitive stimulation therapy, serious games, memory training, olfactory training, and non-invasive brain stimulation (9). Despite the growing interest in non-pharmacological interventions, the strength of evidence varies considerably across different approaches. Cognitive training and multidomain interventions are supported by a relatively substantial body of high-quality evidence, whereas evidence for emerging approaches, such as virtual reality-based training, remains preliminary (10, 11). In addition, existing studies demonstrate considerable heterogeneity in intervention duration, frequency, delivery format, including individual, group-based, and home-based or remote delivery, as well as in the outcome measures used, thereby limiting direct comparisons of intervention effectiveness (12). Most studies have compared a single intervention with usual care, while direct head-to-head randomised evidence among different interventions remains relatively scarce. Consequently, it remains unclear which intervention is most effective for improving cognitive function in older adults, and no definitive hierarchy is currently available to guide clinical decision-making (13). Therefore, this study employed a network meta-analysis to compare the effects of eight non-pharmacological cognitive interventions on cognitive function in older adults with cognitive impairment and to rank their relative effectiveness. The findings are expected to provide evidence-based guidance for selecting appropriate non-pharmacological interventions in the clinical nursing care of older adults with cognitive impairment.

## 2 Methods

This study was registered with the International Prospective Register of Systematic Reviews (PROSPERO), registration number CRD420251011994.

### 2.1 Literature search

The Cochrane Library, PubMed, Embase, Web of Science, China National Knowledge Infrastructure (CNKI), China Biomedical Literature Service, VIP Database, and Wanfang databases were searched to collect randomized controlled trials of cognitive interventions for the treatment of cognitive impairment in elderly patients. The search period ranged from the inception of each database to August 2025. The search strategy combined subject headings and free-text keywords. Search terms include “Cognitive Dysfunction/Cognitive Disorder/Cognitive Impairment/Mild Cognitive Impairment/Cognitive Decline/Mental Deterioration,” “Cognitive Training/Cognitive Rehabilitation/Brain Training/Memory Training,” “Aged/Elderly,” “randomized/controlled trial/randomized/placebo.”

### 2.2 Study selection process

All retrieved records were imported into EndNote X9 software for duplicate removal. Two researchers independently screened the literature. First, titles and abstracts were reviewed to exclude studies that clearly did not meet the inclusion criteria. Then, the full texts of the remaining articles were assessed according to the predefined inclusion and exclusion criteria. Any disagreement between the two researchers was resolved through discussion, and a third researcher was consulted when necessary. The study selection process was mainly based on study type, target population, intervention characteristics, and outcome measures.

### 2.3 Literature inclusion and exclusion criteria

#### 2.3.1 Inclusion criteria

1. Type of study: Randomized controlled trials (RCTs) were included. 2. Study population: Individuals aged 60 years and older, diagnosed with cognitive impairment based on the Mini-Mental State Examination (MMSE) or according to the Diagnostic Guidelines for Cognitive Impairment (14). 3. Intervention: The control group received conventional rehabilitation treatment and care, blank control, or placebo; The intervention group underwent cognitive interventions, which included computerized cognitive training (CCT), integrated cognitive training, virtual reality technology (VR), cognitive stimulation therapy (CST), serious games, memory training, olfactory training (OT), and non-invasive brain stimulation (NIBS). CCT involved cognitive training through conventional computer systems using standardized procedures and interactive tasks to improve specific cognitive abilities. Integrated cognitive training was a multidomain intervention designed to enhance various cognitive functions, including memory, attention, executive function, language, and visuospatial abilities, through systematic exercises. VR provided immersive, controlled experiences that activated brain functions by presenting real challenges in virtual scenarios. CST incorporated components such as exercise, diet, sleep regulation, music therapy, and psychosocial support to enhance cognitive and social functioning. Serious games combined entertainment, education, health care, and treatment through experiential digital games aimed at improving cognitive skills. Memory training focused on enhancing memory using simple and easily implemented techniques for clinical use. OT involved short-term, frequent exposure to various odors to stimulate and improve the function of the olfactory system and cognitive functions. Finally, NIBS utilized external devices to modulate neural activity in the brain non-invasively, without requiring surgical procedures. 4. Outcome indicators: The Mini-Mental State Examination (MMSE) and Montreal Cognitive Assessment (MoCA) were used as outcome indicators.

#### 2.3.2 Exclusion criteria

1. Non-Chinese and non-English literature; 2. studies focusing on cognitive impairment caused by other diseases, such as vascular cognitive impairment, Parkinson’s disease, stroke, or traumatic cognitive impairment; 3. studies with poor quality or obvious data errors; 4. literature for which original data could not be obtained or from which data could not be extracted and converted; 5. conference literature, reviews, dissertations, and non-randomized controlled trials; 6. studies with unclear intervention methods; 7. studies involving combined interventions in which the independent effect of cognitive intervention could not be evaluated, such as those in which the intervention group received additional non-cognitive active interventions, including exercise, nutritional, psychological, or pharmacological interventions, while the control group did not receive the same measures; 8. studies in which pharmacological treatment was used as the main intervention, or in which differences in medication use between groups might affect cognitive outcomes. Studies were not excluded if both groups received the same usual care, basic treatment, or stable medication.

### 2.4 Data extraction

Data were independently extracted by two researchers and cross-checked for accuracy. The extracted information included: 1. basic study information, such as the first author, year of publication, and country; 2. baseline characteristics of the participants, such as sample size and age; 3. intervention and control measures; 4. intervention duration; 5. outcome measures and relevant data. Any disagreement during data extraction was resolved through discussion or consultation with a third researcher.

### 2.5 Quality evaluation

Because all studies included in this review were randomized controlled trials, the Cochrane risk-of-bias tool for randomized trials was used to assess the methodological quality of the included studies. This tool is commonly used in systematic reviews to evaluate the risk of bias in randomized controlled trials, mainly covering random sequence generation, allocation concealment, blinding, completeness of outcome data, selective reporting, and other sources of bias (15, 16). Quality assessment was independently conducted by two researchers, and each domain was judged as “low risk of bias,” “unclear risk of bias,” or “high risk of bias.” Any disagreement was resolved through discussion or consultation with a third researcher.

### 2.6 Statistical analysis

RevMan 5.4 software was used to generate the risk-of-bias graph. Bayesian network meta-analysis was performed using the gemtc package in R software (version 4.5.2) within the RStudio environment (version 2025.09.2 Build 418). For each model, four Markov chains were established, with 20,000 iterations, of which the first 5,000 were used for burn-in. Model convergence was assessed using the potential scale reduction factor (PSRF), with a PSRF value close to 1 indicating satisfactory convergence.

Stata 16.0 software was used to draw the evidence network plot, in which node size reflected sample size and line thickness represented the strength of direct evidence. The node-splitting method was used to assess the consistency between direct and indirect evidence. If the node-splitting results showed no statistically significant difference (*p* ≥ 0.05), the direct and indirect evidence were considered to be consistent, and a consistency model was used for analysis. If a statistically significant difference was observed (*p* < 0.05), inconsistency was considered to exist, and an inconsistency model was used, with further exploration of potential sources of inconsistency. Heterogeneity was mainly assessed using *τ*^2^, supplemented by *I*^2^. In general, *I*^2^ ≤ 50% indicated low heterogeneity or good homogeneity among studies, whereas *I*^2^ > 50% suggested substantial heterogeneity, in which case a random-effects model was preferred. When *τ*^2^ or *I*^2^ indicated obvious heterogeneity, sensitivity analysis or subgroup analysis was further performed to explore potential sources of heterogeneity. The surface under the cumulative ranking curve (SUCRA) was used to quantify the ranking probability of each intervention. A higher SUCRA value indicated a greater probability that the intervention was the optimal option. Funnel plots were used to assess publication bias. The outcome measures in this study were continuous variables, and mean difference (MD) with 95% CI was used as the effect size. A value of *p* < 0.05 was considered statistically significant.

## 3 Results

### 3.1 Literature search process and results

**Figure 1:**
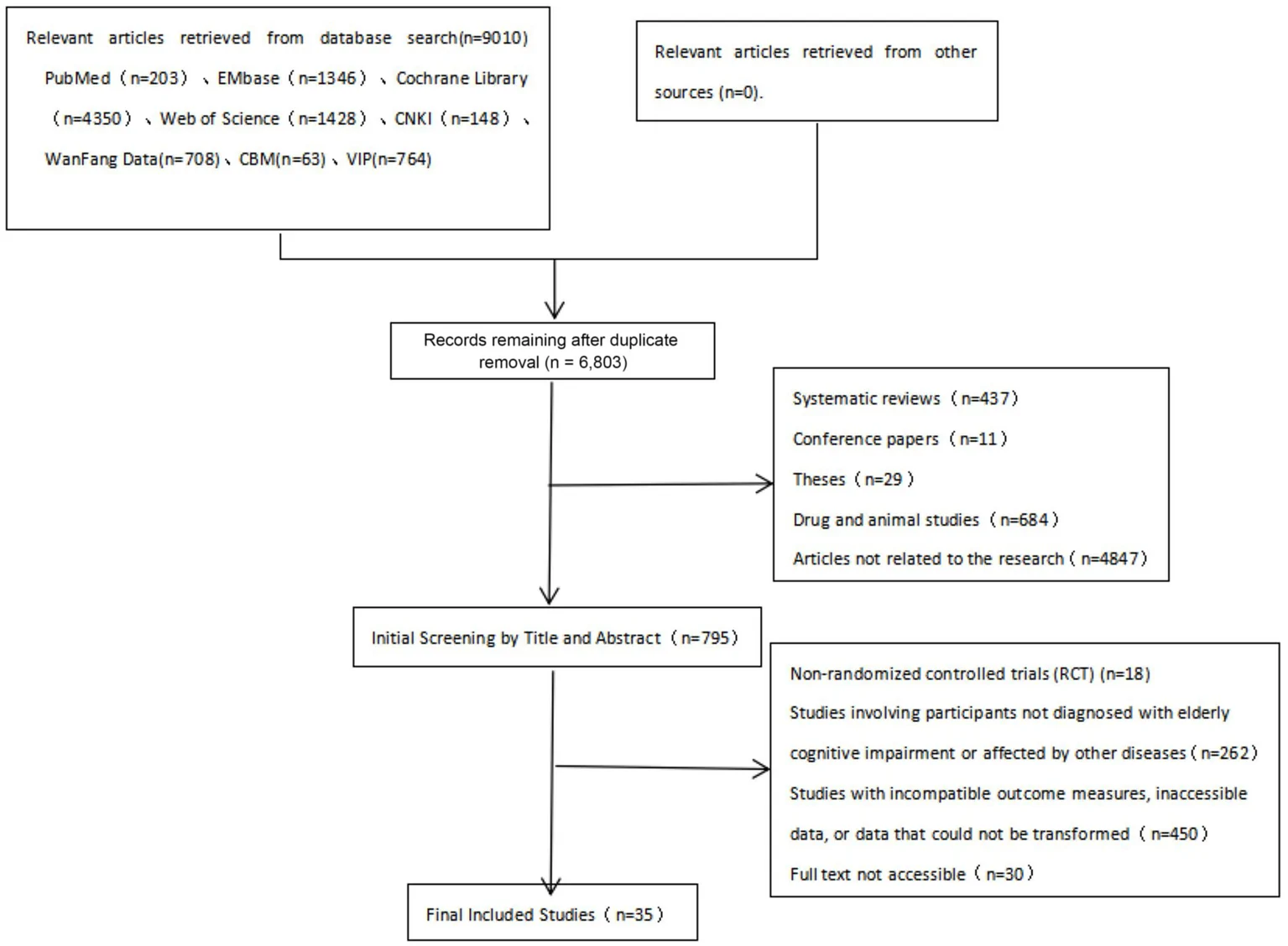
Process of the systematic review.

The search of related databases initially retrieved 9,010 pieces of literature, including PubMed (*n* = 203), Embase (*n* = 1,346), Cochrane Library (*n* = 4,350), Web of Science (*n* = 1,428), CNKI (*n* = 148), WanFang Data (*n* = 708), CBM (*n* = 63), and VIP (*n* = 764). After screening, 35 documents were finally included. The literature screening process is shown in Figure 1.

### 3.2 Basic characteristics of the included studies

A total of thirty-five randomized controlled trials were included in the network meta-analysis (Table 1), comprising 2,316 elderly patients with cognitive impairment of both sexes. The treatment group included cognitive interventions such as computerized cognitive training (CCT), integrated cognitive training, virtual reality (VR), cognitive stimulation therapy (CST), serious games, memory training, olfactory training (OT), and non-invasive brain stimulation (NIBS).

**Table 1:** Basic characteristics of the included literature.

Study	Country	Age (T/C)	Sample size (T/C)	Intervention/control measures	Treatment time	Outcome
Zhou et al. (17)	China	80.58 ± 4.76/80.03 ± 4.36	50/52	CCT/usual care	3 months	①
Li et al. (18)	China	70.63 ± 6.32/71.62 ± 5.27	30/29	CCT/usual care	5 weeks	②
Guo et al. (19)	China	74.76 ± 2.31/75.31 ± 2.02	124/125	CCT/usual care	4 weeks	①②
Wu et al. (20)	China	67.68 ± 5.83/65.52 ± 5.55	25/25	CCT/usual care	8 weeks	②
Weng et al. (21)	China	>60	33/29	CCT/usual care	3 months	②
Kang et al. (22)	Korea	74.36 ± 6.73/75.08 ± 4.52	15/12	CCT/CST	8 weeks	①②
Carvalho et al. (23)	Brazil	74.24 ± 8.27/75.15 ± 6.96	29/26	CCT/serious games	10 h	②
Baik et al. (24)	Korea	67.08 ± 7.93/65.64 ± 8.54	25/25	CCT/usual care	9 weeks	②
Huang et al. (25)	China	71.91 ± 8.50	56/50	integrated cognitive training/usual care	48 weeks	①②
Hu et al. (26)	China	63.48 ± 2.35/62.92 ± 2.69	50/52	integrated cognitive training/usual care	12 weeks	②
Gu et al. (27)	China	>60	59/59	integrated cognitive training/usual care	4 months	②
Fan et al. (28)	China	67.13 ± 1.93/65.46 ± 1.58	37/38	integrated cognitive training/usual care	6 months	①②
Peng et al. (29)	China	68.39 ± 4.21/68.69 ± 4.26	70/70	integrated cognitive training/usual care	6 months	②
Rojas et al. (30)	Argentina	72.00 ± 14.29/76.93 ± 7.05	15/15	integrated cognitive training/usual care	6 months	①
Giuli et al. (31)	Italy	76.00 ± 6.30/76.50 ± 5.70	48/49	integrated cognitive training/usual care	10 weeks	①
Barekatain et al. (32)	Iran	66.20 ± 5.50/65.70 ± 4.70	17/19	integrated cognitive training/usual care	8 weeks	①
Thapa et al. (33)	Korea	72.60 ± 5.40/72.70 ± 5.60	34/34	VR/usual care	8 weeks	①
Park et al. (34)	Korea	71.80 ± 6.61/69.45 ± 7.45	10/11	VR/usual care	12 weeks	①
Huang et al. (35)	China	69.54 ± 5.17/71.12 ± 5.28	49/48	VR/usual care	12 weeks	①②
Gu et al. (36)	China	72.36 ± 5.47/73.52 ± 5.64	50/50	CST/usual care	6 months	①
Mao et al. (37)	China	67.30 ± 4.48/66.83 ± 4.19	30/30	CST/usual care	1 year	①
Justo-Henriques et al (38).	Portugal	78.53 ± 7.82/79.21 ± 7.37	30/29	CST/usual care	47 weeks	①②
Cove et al. (39)	UK	76.80 ± 6.62/77.80 ± 7.47	24/23	CST/usual care	14 weeks	①
Carcelén-Fraile et al. (40)	Spain	75.36 ± 3.74/74.80 ± 3.92	36/35	CST/usual care	12 weeks	①②
Sung et al. (41)	China-Taiwan	83.50 ± 6.36/81.00 ± 6.41	36/36	serious games/CST	8 weeks	①
Savulich et al. (42)	UK	75.20 ± 7.40/76.90 ± 8.30	21/21	serious games/usual care	4 weeks	①
Lim et al. (43)	Korea	75.42 ± 5.74/73.33 ± 17.52	12/12	serious games/usual care	4 weeks	①②
Goumopoulos et al. (44)	Greece	74.00 ± 4.50/72.00 ± 3.80	11/10	serious games/usual care	12 weeks	②
Zhang et al. (45)	China	60–75	21/21	memory training/usual care	3 months	①
Yang et al. (46)	China-Taiwan	75.40 ± 6.60/81.70 ± 7.20	33/33	memory training/CST	12 weeks	①
Chen et al. (47)	Germany	72.70 ± 4.90/70.60 ± 7.00	17/16	OT/Odorless training	4 months	①
Haehner et al. (48)	Germany	71.40 ± 5.10/70.30 ± 7.30	19/18	OT/Odorless training	4 months	①
Gu et al. (49)	China	63.20 ± 6.98/65.15 ± 6.16	20/20	NIBS/usual care	4 weeks	②
Im et al. (50)	Korea	71.90 ± 9.20/74.90 ± 5.00	11/7	NIBS/usual care	6 months	①
Wang et al. (51)	China	65.60 ± 4.20/68.40 ± 5.10	20/20	NIBS/usual care	10d	①②

T, intervention; C, control measures; ① MMSE; ② MoCA.

### 3.3 Results of risk of bias evaluation

**Figure 2:**
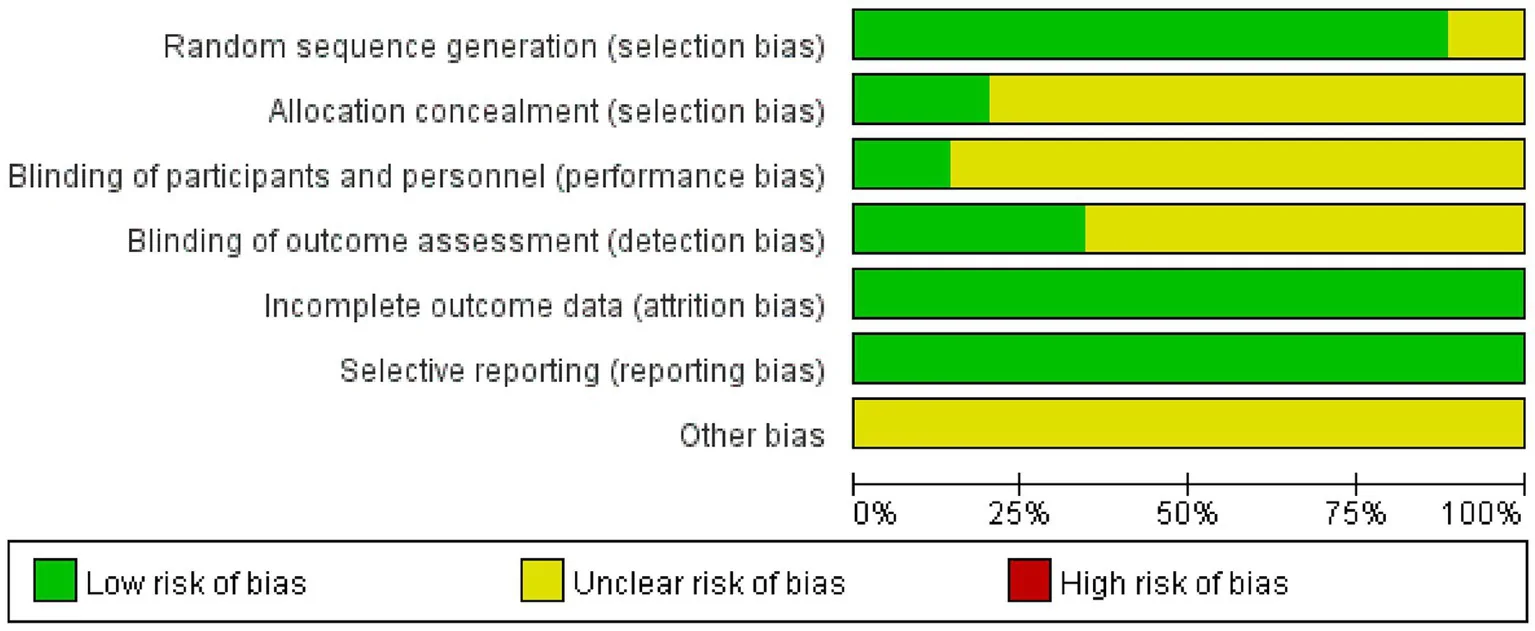
Evaluation chart of the risk of bias in the included literature.

Thirty-five RCTs were included in this analysis. Among these, 24 RCTs (18, 20, 22–26, 29, 31–36, 39–43, 45, 47, 49–51) reported specific methods for random sequence generation. Eight RCTs (23, 32, 38–40, 46, 49) provided specific details regarding the implementation of allocation concealment, while 8 studies (20, 24, 32, 38, 39, 43, 46, 48) were single-blinded, and 4 studies (23, 41, 47, 50) were double-blinded. The remaining studies did not mention allocation concealment or blinding methods. All included studies described loss to follow-up, demonstrating good data completeness. No selective reporting or other sources of bias were identified (Figure 2).

### 3.4 Evidence network

#### 3.4.1 Evidence network analysis

**Figure 3:**
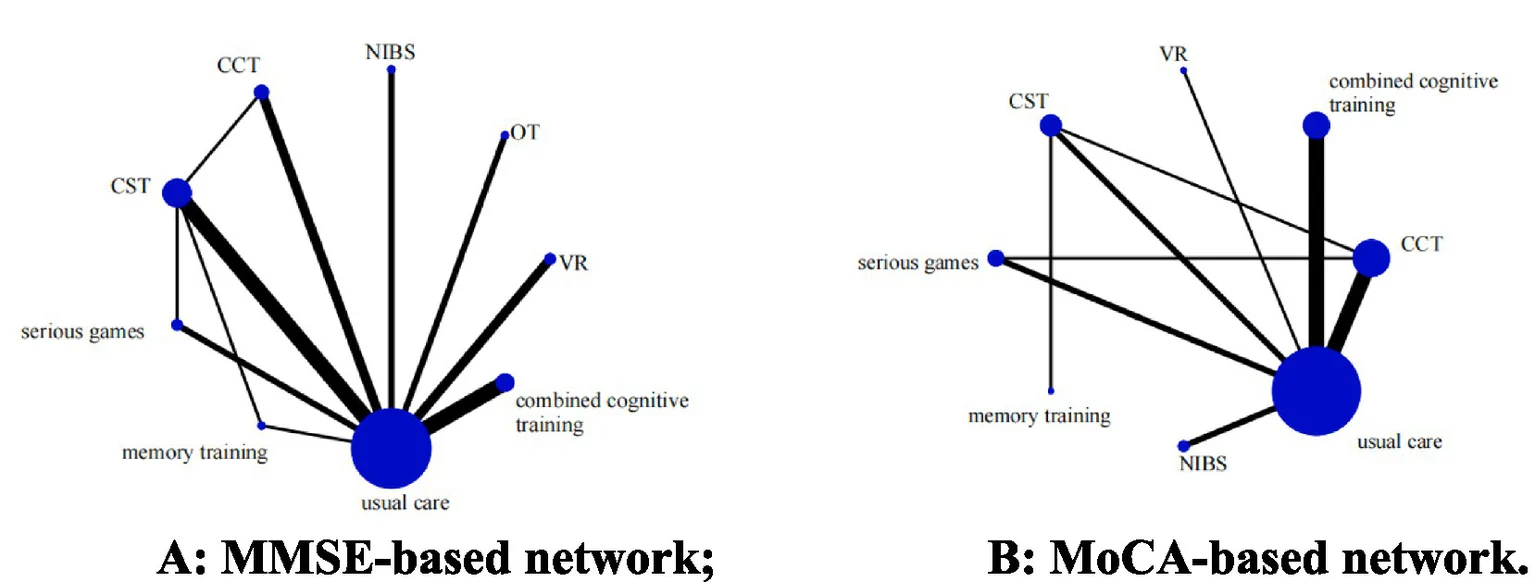
Evidence networks for different cognitive function outcome measures: **(A)** MMSE-based network; **(B)** MoCA-based network. Node size is proportional to the cumulative sample size, and line thickness is proportional to the number of studies providing direct comparisons.

The evidence networks for the two cognitive outcome measures are presented separately in Figure 3. Figure 3A shows the evidence network based on Mini-Mental State Examination (MMSE) scores, whereas Figure 3B shows the network based on Montreal Cognitive Assessment (MoCA) scores. Because the included studies did not report the same outcome measures consistently, separate evidence networks were formed for the MMSE and MoCA outcomes. In each network, node size is proportional to the cumulative sample size for the corresponding intervention, while line thickness is proportional to the number of studies providing direct comparisons between two interventions. Usual care served as the main comparator in both networks, and the relative effects of some interventions were estimated primarily through indirect evidence.

#### 3.4.2 Assessment of heterogeneity and inconsistency

For the MMSE outcome, the overall assessment of heterogeneity across the network yielded a *τ*^2^ of 0.8318 and an *I*^2^ of 73.3% (95% CI, 58.0–83.1%), indicating substantial between-study heterogeneity. The test for inconsistency between study designs was not statistically significant (*p* = 0.5829). For the MoCA outcome, the overall network heterogeneity was also substantial, with a *τ*^2^ of 1.5708 and an *I*^2^ of 74.4% (95% CI, 56.7–84.9%). No statistically significant design-by-treatment interaction was detected (*p* = 0.6693). The node-splitting analysis showed no statistically significant differences between direct and indirect evidence for any comparison (all *p* > 0.05), suggesting no evidence of local inconsistency. Based on the results of both global and local inconsistency assessments, a random-effects consistency model was used for the subsequent network meta-analysis.

### 3.5 Results of the network meta-analysis

#### 3.5.1 MMSE score

**Figure 4:**
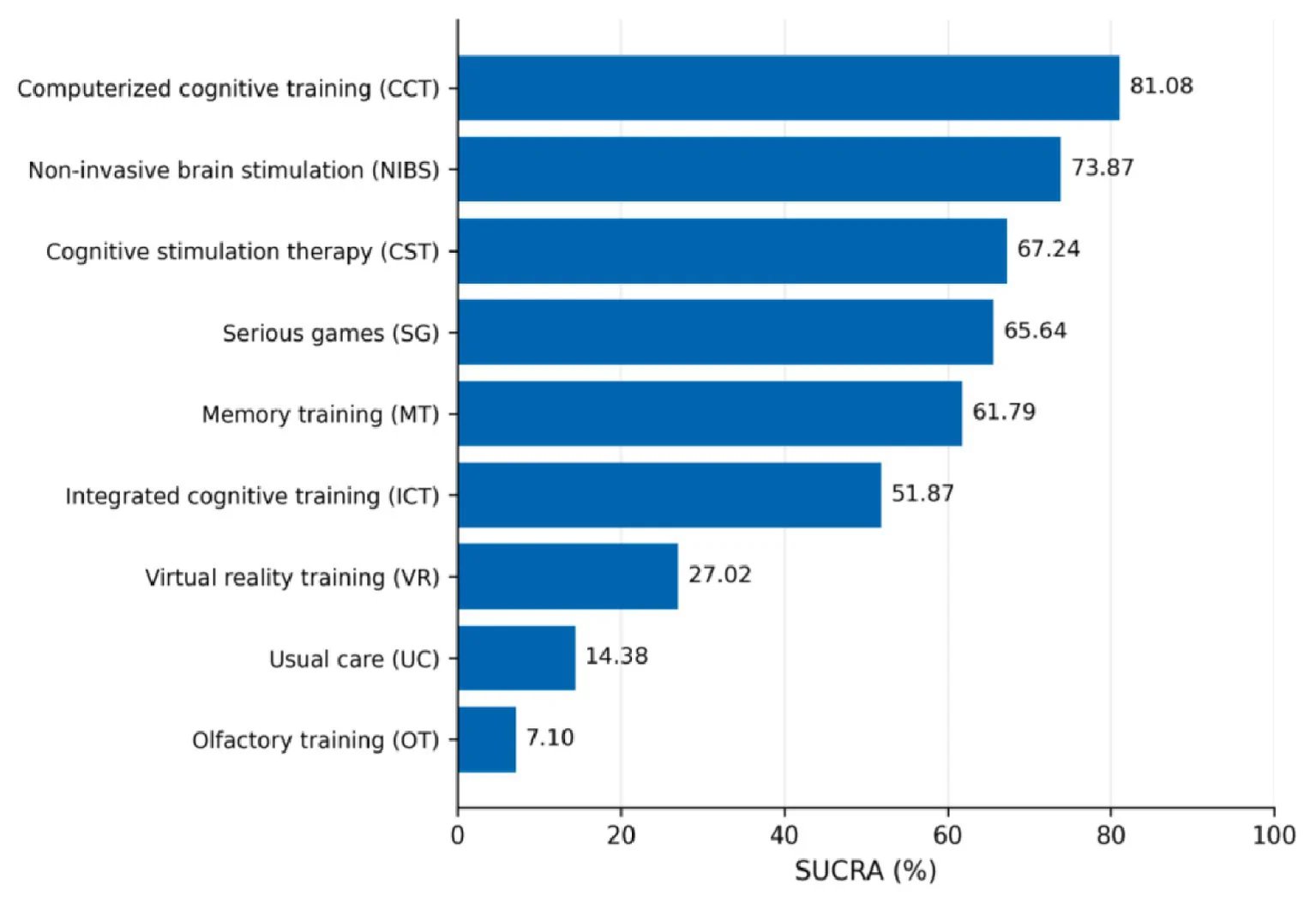
SUCRA ranking of cognitive interventions based on MMSE scores. A higher SUCRA value indicates a greater probability that an intervention ranks among the more effective options.

The results of the network meta-analysis (Table 2) indicated that CCT, integrated cognitive training, CST, and serious games outperformed usual care, while CCT, CST, and serious games were superior to olfactory training. Eight interventions were compared; however, no statistically significant differences were found among them. The cumulative probability ranking of interventions, from highest to lowest, was as follows: CCT (SUCRA = 81.08%), NIBS (SUCRA = 73.87%), CST (SUCRA = 67.24%), serious games (SUCRA = 65.64%), memory training (SUCRA = 61.79%), integrated cognitive training (SUCRA = 51.87%), VR (SUCRA = 27.02%), usual care (SUCRA = 14.38%), and olfactory training (SUCRA = 7.1%) (Figure 4).

**Table 2:** League table of network meta-analysis results based on MMSE scores (MD, 95% CI).

CCT	ICT	VR	CST	SG	MT	OT	NIBS	UC
CCT								
0.87 (−0.75, 2.48)	ICT							
1.74 (−0.21, 3.72)	0.87 (−0.95, 2.72)	VR						
0.45 (−1.05, 1.89)	−0.42 (−1.83, 0.94)	−1.29 (−3.12, 0.46)	CST					
0.47 (−1.43, 2.35)	−0.40 (−2.20, 1.37)	−1.27 (−3.39, 0.82)	0.02 (−1.43, 1.52)	SG				
0.57 (−1.51, 2.62)	−0.29 (−2.30, 1.66)	−1.16 (−3.48, 1.08)	0.13 (−1.57, 1.83)	0.11 (−2.05, 2.23)	MT			
2.74 (0.62, 4.88)	1.88 (−0.13, 3.88)	1.00 (−1.32, 3.30)	2.30 (0.35, 4.28)	2.27 (0.04, 4.53)	2.17 (−0.22, 4.61)	OT		
0.07 (−2.56, 2.66)	−0.80 (−3.35, 1.71)	−1.67 (−4.46, 1.05)	−0.37 (−2.86, 2.10)	−0.40 (−3.12, 2.3)	−0.50 (−3.35, 2.35)	−2.67 (−5.56, 0.18)	NIBS	
2.14 (0.90, 3.39)	1.28 (0.24, 2.31)	0.41 (−1.13, 1.91)	1.70 (0.80, 2.65)	1.67 (0.23, 3.13)	1.57 (−0.09, 3.28)	−0.60 (−2.33, 1.13)	2.07 (−0.21, 4.39)	UC

Data are presented as mean differences (MDs) with 95% confidence intervals (CIs), and only the lower triangular portion of the league table is shown. Each cell represents the effect estimate for the intervention in the corresponding column relative to the intervention in the corresponding row. Positive values favor the column intervention, whereas negative values favor the row intervention. A difference is considered statistically significant when the 95% CI does not include zero.

#### 3.5.2 MoCA score

**Figure 5:**
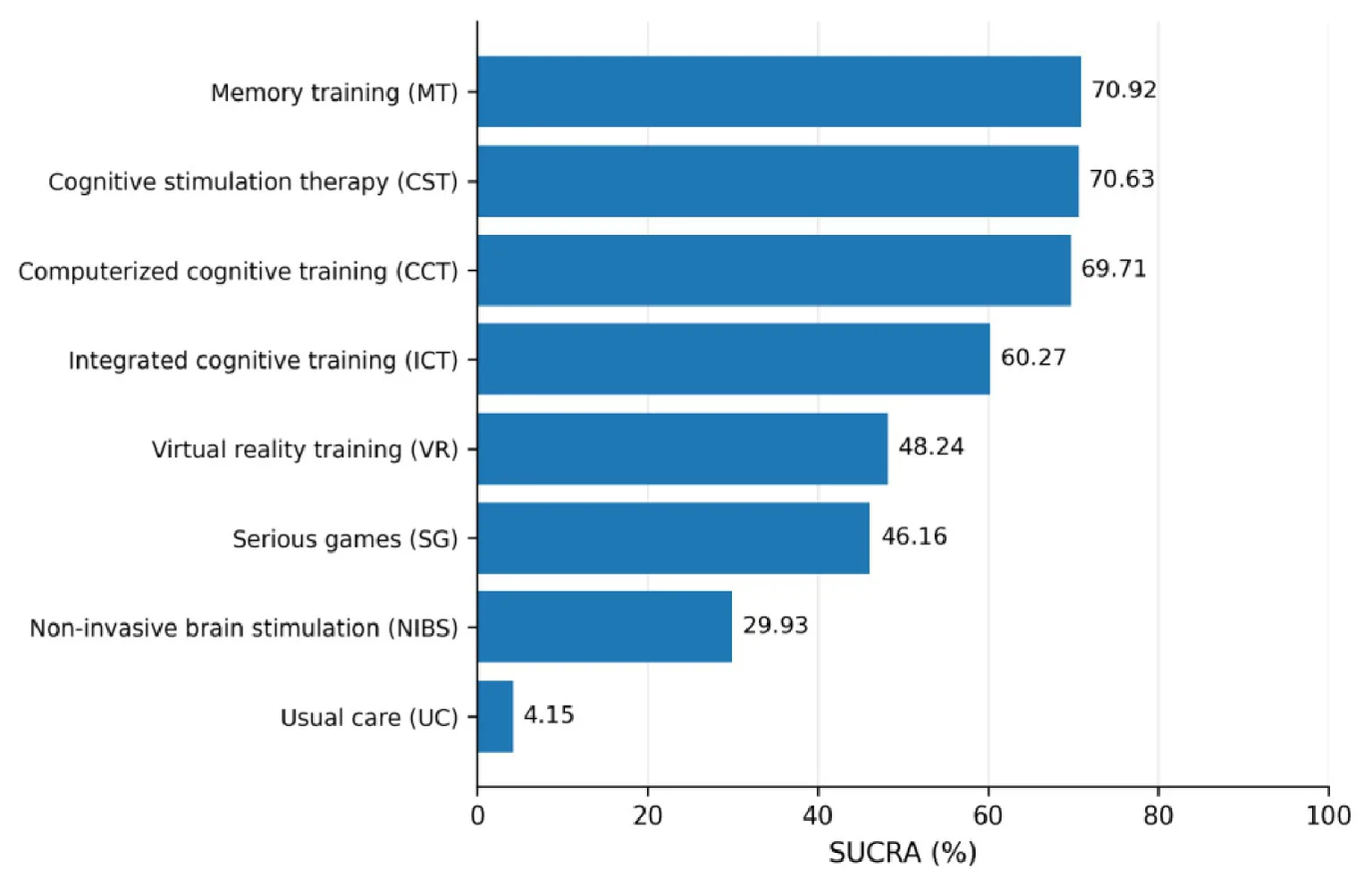
SUCRA ranking of cognitive interventions based on MoCA scores.

The net meta-analysis (Table 3) indicated that CCT, integrated cognitive training, and CST outperformed usual care; however, no statistically significant differences were found among the seven interventions when compared with each other. The cumulative probability ranking of interventions, from highest to lowest, was as follows: memory training (SUCRA = 70.92%), CST (SUCRA = 70.63%), CCT (SUCRA = 69.71%), integrated cognitive training (SUCRA = 60.27%), VR (SUCRA = 48.24%)serious games (SUCRA = 46.16%), NIBS (SUCRA = 29.93%), and usual care (SUCRA = 4.15%) (Figure 5).

**Table 3:** League table of network meta−analysis results based on MoCA scores (MD, 95% CI).

CCT	ICT	VR	CST	SG	MT	NIBS	UC
CCT							
0.32 (−1.67, 2.28)	ICT						
0.86 (−2.79, 4.58)	0.54 (−3.13, 4.30)	VR					
−0.13 (−2.59, 2.22)	−0.44 (−3.09, 2.12)	−0.98 (−5.13, 3.00)	CST				
0.90 (−1.48, 3.43)	0.59 (−2.18, 3.54)	0.03 (−4.11, 4.34)	1.02 (−2.07, 4.39)	SG			
−0.40 (−4.76, 3.84)	−0.71 (−5.17, 3.64)	−1.27 (−6.73, 4.04)	−0.27 (−3.83, 3.27)	−1.30 (−6.27, 3.37)	MT		
1.71 (−1.13, 4.53)	1.39 (−1.48, 4.28)	0.85 (−3.43, 5.05)	1.84 (−1.40, 5.17)	0.81 (−2.77, 4.2)	2.12 (−2.69, 7.00)	NIBS	
3.13 (1.79, 4.50)	2.81 (1.41, 4.28)	2.27 (−1.18, 5.69)	3.26 (1.16, 5.50)	2.23 (−0.28, 4.63)	3.54 (−0.57, 7.77)	1.42 (−1.04, 3.94)	UC

Data are presented as mean differences (MDs) with 95% confidence intervals (CIs), and only the lower triangular portion of the league table is shown. Each cell represents the effect estimate for the intervention in the corresponding column relative to the intervention in the corresponding row. Positive values favor the column intervention, whereas negative values favor the row intervention. A difference is considered statistically significant when the 95% CI does not include zero.

A higher SUCRA value indicates a greater probability that an intervention ranks among the more effective options.

### 3.6 Publication bias

**Figure 6:**
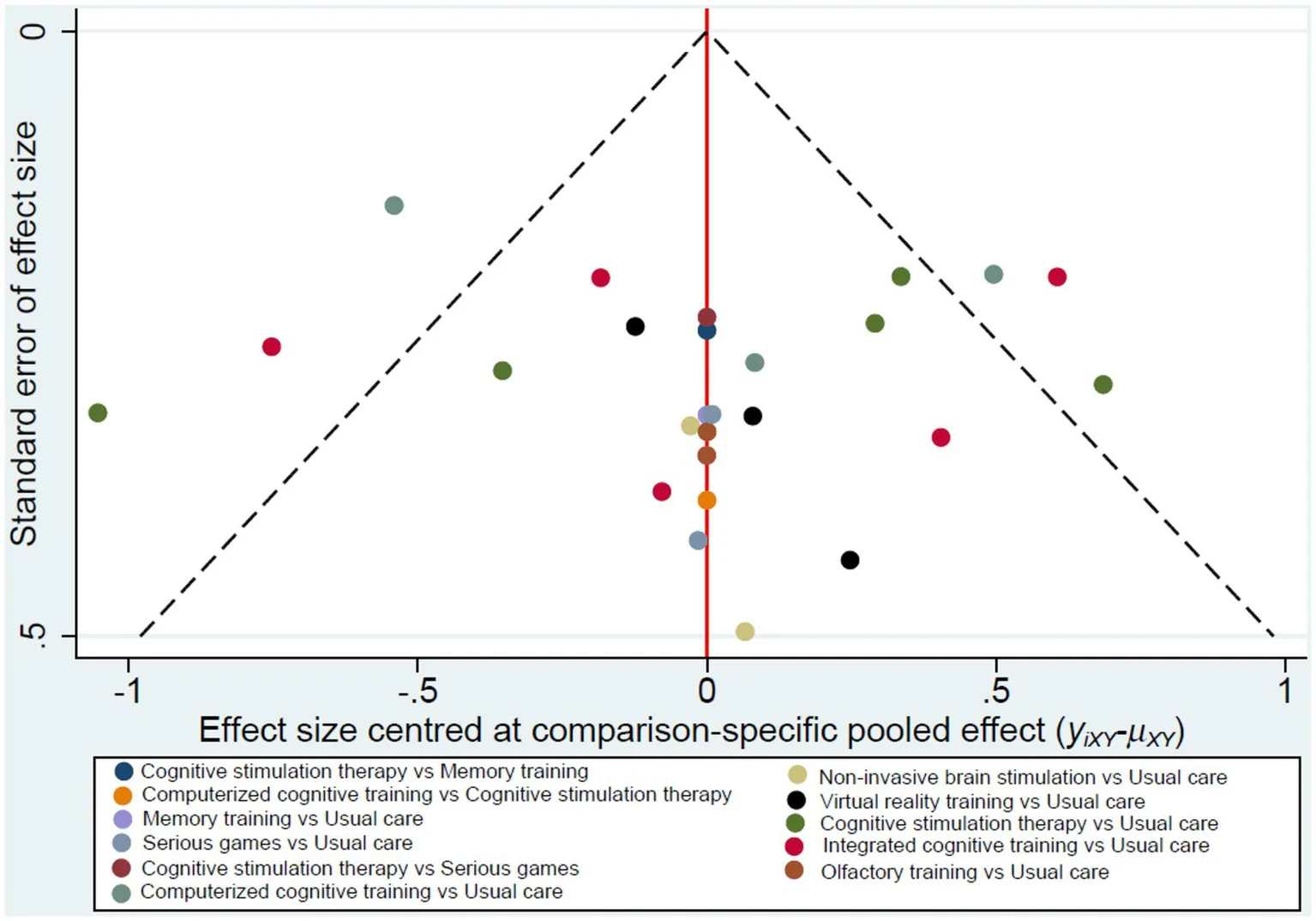
Funnel plot of publication bias for the Mini-Mental State Examination.

**Figure 7:**
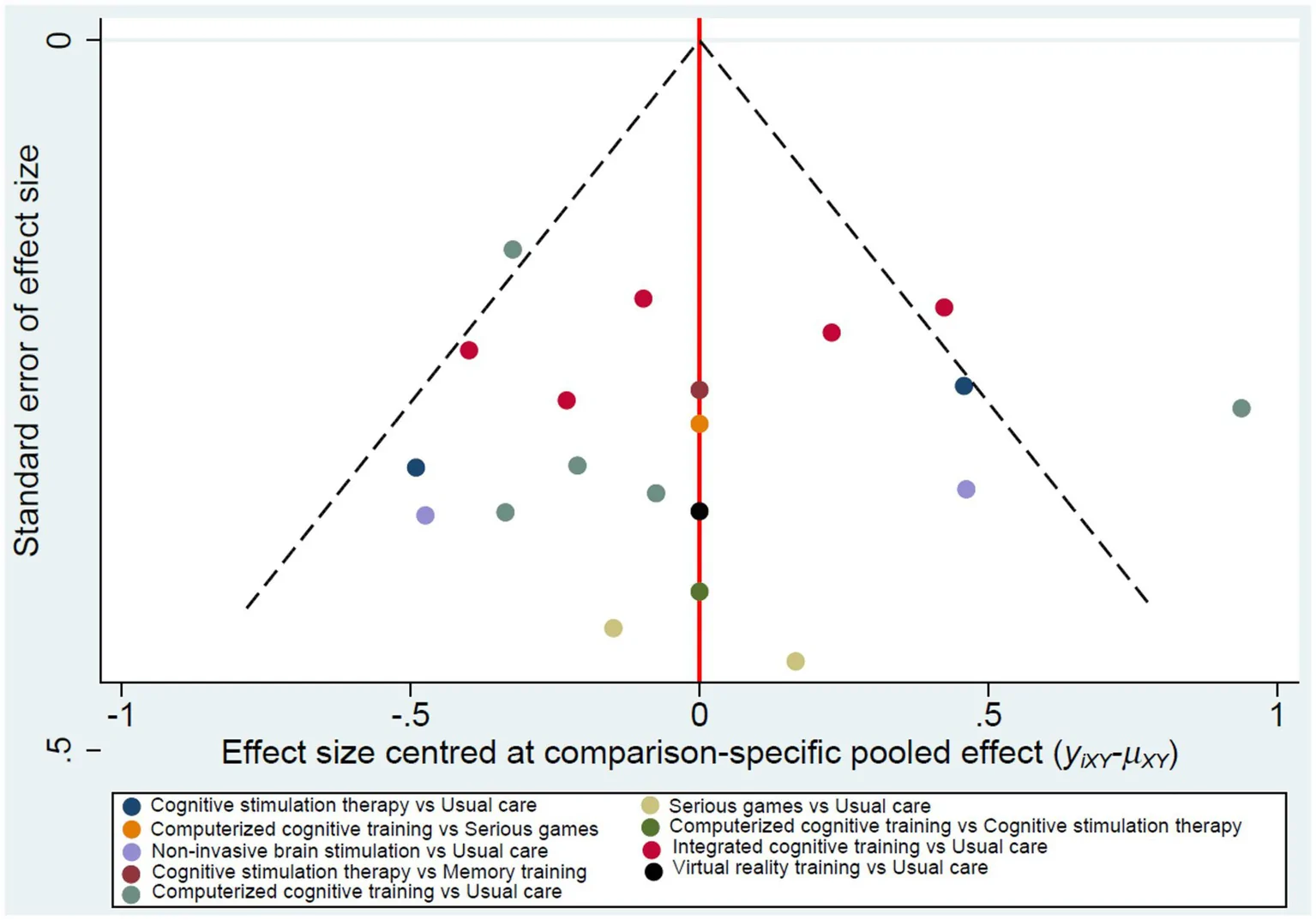
Funnel plot of publication bias for the Montreal Cognitive Assessment (MoCA).

The funnel plot of the included studies was symmetrically distributed around the combined effect sizes, suggesting a relatively favorable distribution of studies and a low risk of publication bias. The funnel plot is presented in Figures 6, 7.

## 4 Discussion

Cognitive intervention is an important non-pharmacological approach for improving cognitive function in older adults with cognitive impairment (52), and its effects may be associated with the maintenance of neuroplasticity and cognitive reserve (53, 54). These theoretical frameworks suggest that targeted and sustained training can activate the central nervous system, promote the reorganization of neural networks in adjacent healthy brain regions, and compensate for impaired functions. Such training may significantly enhance interregional brain connectivity, particularly white matter integrity, and optimize the functioning of higher-order cognitive networks (55). Neuroscientific studies have further demonstrated that aging and neurodegenerative processes are often accompanied by reduced cholinergic neurotransmission (56), while the functional integrity of the dorsal cingulate cortex plays a direct role in regulating core cognitive domains, including executive function and episodic memory (57).

In the present study, CCT ranked first in the SUCRA hierarchy for the MMSE network and third for the MoCA network. For both outcomes, CCT was more effective than usual care, suggesting a relatively consistent benefit in improving global cognitive function. This advantage may be attributable to the standardized, feedback-driven, and adaptive nature of CCT. Computerized systems can deliver training repeatedly using relatively consistent task procedures and scoring criteria, thereby reducing variability in intervention implementation (58). Participants can also receive immediate feedback on task performance (59), while some systems dynamically adjust task difficulty according to individual performance, allowing the training demands to remain aligned with the participant’s current cognitive capacity (60). In addition, some CCT programs present training tasks through visual and auditory modalities, enabling simultaneous stimulation of multiple cognitive domains, including attention, memory, and executive function (61, 62). From a clinical implementation perspective, CCT is well suited to individualized and home-based delivery. However, its application should take into account access to appropriate devices, patients’ digital literacy, visual and auditory functioning, and adherence to sustained training. Caregiver or professional support should also be provided when necessary.

Memory training (MT) ranked first in the SUCRA hierarchy for the MoCA network; however, the interval estimate for its relative effect versus usual care crossed the line of no effect, and therefore a definitive advantage cannot be established. The finding of a high ranking without statistical significance is not contradictory. SUCRA reflects the probability that an intervention will achieve a higher rank within the current evidence network, whereas statistical significance also depends on sample size and the precision of the effect estimate. In the present network, the evidence for MT was limited, the sample size was small, and most comparisons with other interventions relied on indirect evidence. Consequently, the stability of its ranking was limited, resulting in a relatively high rank but wide interval estimates. A longitudinal study reported that the delayed-recall score on the Rey Auditory Verbal Learning Test (RAVLT) independently predicted progression to Alzheimer’s disease dementia, with each one-point increase in delayed recall associated with a reduced risk of progression (OR = 0.84, 95% CI: 0.76–0.92) (63). This finding indicates that delayed recall is an important marker for identifying individuals at risk of cognitive deterioration; however, predictive evidence does not directly demonstrate that MT has greater therapeutic efficacy. Therefore, memory should be regarded as one important cognitive domain targeted by cognitive interventions rather than the sole therapeutic target. At present, MT should not be recommended as the preferred intervention solely on the basis of its SUCRA ranking. Large-scale randomized controlled trials with active comparators are still needed to verify its effects on global cognition and activities of daily living.

The relative ranking of NIBS differed between the MMSE and MoCA networks, which may reflect differences in the domains emphasized by the two instruments, as well as variations in stimulation targets, intensity, treatment duration, and participant characteristics across the original studies. The MMSE primarily assesses global cognitive function, including orientation, memory, attention, and language, whereas the MoCA is more sensitive to mild cognitive impairment involving executive function, cognitive flexibility, and abstract thinking (64). Therefore, differences in ranking between the two networks should not be interpreted as evidence that NIBS is more effective for a specific type of cognitive function. Future studies should incorporate domain-specific cognitive measures to further clarify the effects of tDCS and rTMS on memory, attention, and executive function.

Cognitive stimulation therapy (CST) has demonstrated efficacy in both assessments. A range of intervention modalities, including musical memory, daily task simulation, and orientation training, engage multiple cognitive domains, which in turn enhances engagement and promotes neural circuit plasticity, thereby strengthening executive function and attention (38). Moreover, Carcelén-Fraile et al. (40) showed that CST not only improves cognitive function but also positively influences patients’ mood and quality of life. However, the frequency and form of intervention significantly affect the outcomes. Cove (39) and colleagues noted that weekly CST did not provide sufficient stimulation to counteract neurodegenerative processes and did not yield any improvements in cognitive indicators. In contrast, individualized interventions with higher frequency combine psychological support with the integration of lifestyle changes. These interventions have been shown to enhance both self-efficacy and social participation in patients. In clinical practice, structured and regularly scheduled group-based cognitive stimulation therapy (CST) may be prioritized when patients can tolerate the intervention and institutional resources permit. The intervention should be individualized according to patients’ cognitive status, sensory functioning, and capacity for participation. For those with transportation difficulties or who are unable to attend group sessions, individualized or remotely delivered formats may be considered; however, their optimal frequency and long-term maintenance strategies require further investigation.

It should be noted that repeated administration of cognitive assessments such as the MMSE or MoCA may produce practice effects, as participants become more familiar with the testing procedures, item formats, and response strategies. This may lead to higher scores at follow-up and, to some extent, an overestimation of the intervention effect (65). Most studies included in the present analysis were randomized controlled trials; therefore, both intervention and control groups may have become familiar with the scales through repeated assessment, allowing general practice effects to be controlled to some extent in between-group comparisons. However, because some tasks used in CCT and memory training are similar to items included in these cognitive scales, the possibility that task familiarity contributed to improved follow-up scores cannot be excluded. Accordingly, increases in scale scores should not be interpreted entirely as true improvements in global cognitive function. Future studies should use active control groups, parallel versions of cognitive scales, and blinded outcome assessment, while also incorporating domain-specific cognitive measures and activities of daily living to distinguish genuine cognitive improvement from improvements attributable solely to test familiarity.

Future research should further investigate the combined effects of cognitive interventions with physical exercise, sleep management, and nutritional strategies. Physical exercise may improve cognitive function by enhancing cerebral blood flow, promoting the release of brain-derived neurotrophic factor, increasing synaptic plasticity, and reducing inflammation and oxidative stress (66). Adequate sleep may help maintain glymphatic system function and multimodal brain network connectivity, whereas poor sleep quality may disrupt these neural processes and adversely affect memory function (67). In addition, a balanced diet and improved nutritional status may contribute to the maintenance of cognitive function in older adults. Longitudinal studies have shown that greater adherence to the Mediterranean diet is associated with better cognitive performance in older adults (68). Therefore, multidomain interventions may be more consistent with the complex, multifactorial nature of cognitive impairment in older adults than cognitive training alone; however, their optimal components, sequencing, and target populations require further investigation.

In addition, the long-term durability of the effects of different cognitive interventions remains uncertain. Intervention and follow-up periods in existing studies have generally been short, making it difficult to determine whether cognitive benefits are sustained over time or whether long-term effects differ across interventions (13). Although extending the intervention period may increase the cumulative training dose, sustained participation may be hindered by operational difficulties, unsatisfactory training experiences, delayed perception of benefits, financial concerns, and insufficient family support, potentially resulting in reduced adherence or discontinuation (69). Future studies should further determine the minimum effective dose, duration of maintained effects, and optimal frequency of booster sessions for different interventions, while comprehensively evaluating adherence, cost, and caregiver burden to identify more sustainable intervention models.

## 5 Conclusion

This study used a Bayesian network meta-analysis to compare the relative effects of multiple cognitive interventions on cognitive function in older adults with cognitive impairment. The results showed that computerized cognitive training (CCT) and cognitive stimulation therapy (CST) demonstrated relatively consistent cognitive benefits across the evidence networks based on the MMSE and MoCA, while integrated cognitive training and serious games also showed potential clinical value. Although memory training and non-invasive brain stimulation (NIBS) achieved relatively high SUCRA rankings in some evidence networks, their differences from usual care or other interventions were not consistently statistically significant. These findings should therefore be interpreted cautiously in conjunction with the effect estimates, credible intervals, and certainty of the evidence. Given the small sample sizes of some included studies, variations in participant characteristics, intervention protocols, and follow-up durations, as well as the potential influence of practice effects, inadequate blinding, and language bias, the present findings require further confirmation in large-scale, high-quality randomized controlled trials with long-term follow-up. In clinical practice, individualized cognitive interventions should be selected by comprehensively considering patient characteristics, intervention accessibility, safety, ability to engage with or operate the intervention, and adherence.

## Data Availability

The original contributions presented in the study are included in the article; further inquiries can be directed to the corresponding author.
